# Human cytomegalovirus replication induces endothelial cell interleukin-11

**DOI:** 10.1016/j.cyto.2018.05.018

**Published:** 2018-11

**Authors:** K.L.R. Gustafsson, T. Renné, C. Söderberg-Naucler, L.M. Butler

**Affiliations:** a Department of Medicine, Karolinska Institute, SE-171 76 Stockholm, Sweden; b Institute for Clinical Chemistry and Laboratory Medicine, University Medical Centre Hamburg-Eppendorf, D-20246 Hamburg, Germany

**Keywords:** Interleukin 11, Interleukin 6, Cytomegalovirus, Endothelial cell

## Abstract

Endothelial cells (EC) are critical sites of human cytomegalovirus (hCMV) infection *in vivo*. Infection can induce the production of various EC cytokines, such as interleukin (IL-)6, which can have autocrine and/or paracrine effector functions. Here, we report that hCMV induces the production of EC IL-11, a relatively understudied member of the IL-6-type cytokine family. We detail temporal EC IL-11 translation and protein secretion dynamics in response to hCMV infection, and reveal distinct differences compared to EC IL-6. Viral replication had markedly opposing effects on the regulation of these closely related cytokines, representing a major driving force behind IL-11 production, whilst concurrently suppressing IL-6 expression. This is the first report of any biological agent that stimulates EC IL-11 production.

## Highlights

• Cytomegalovirus induces endothelial cell interleukin-11 secretion.
• Viral replication drives interleukin-11 upregulation at the transcriptional level.
• First report of any biological agent that induces endothelial cell IL-11 production.

## 1 Introduction

Endothelial cells (EC) line blood vessels and are key regulators of vascular integrity, haemostasis and inflammation, and are critical sites of human cytomegalovirus (hCMV) infection *in vivo* [9]. hCMV infection of EC can induce the expression of various interleukins (IL-) [11], the most well studied of which is IL-6, which signals through the gp130 receptor in combination with the IL-6R subunit, to induce signalling through the mitogen-activated protein kinase (MAPK)-cascade and the Janus kinase/signal transducer and activator of transcription (Jak/STAT) pathway [6]. Such pathways induce the expression of EC genes involved in angiogenesis, invasion and inflammation, which are correspondingly modified following hCMV infection e.g. [2]. The IL-6-type cytokine family includes IL-11, which also signals through gp130, but in combination with the IL-11Rα subunit. Considerably less is known about IL-11 regulation and function, but it has a well-established role in haematopoiesis [15] and emerging importance in various pathologies, such as fibrosis [16]. However, currently there are no known physiological or pathological inducers of EC IL-11. Here, we report that hCMV infection strongly induces EC IL-11, and document its temporal expression dynamics compared to the induction of IL-6. Our findings demonstrate markedly differential regulation of these EC cytokines over the hCMV life cycle. The finding that EC can produce IL-11 in response to hCMV infection, has significance from the perspective of both EC biology and viral pathogenesis.

## 2 Results and discussion

**Fig. 1:**
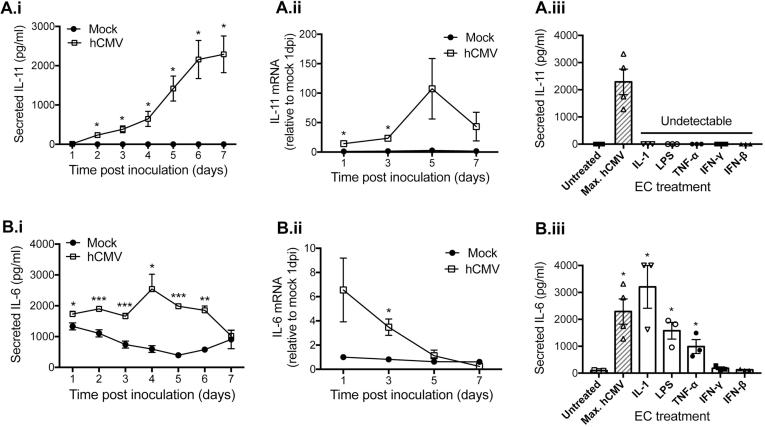
Human cytomegalovirus induces the production of EC IL-11 and IL-6. EC were mock- or hCMV-inoculated and supernatants were collected from each 24 h period for 7 days. EC lysates were harvested at 1, 3, 5 or 7-day post inoculation (dpi). Secreted (A.i) IL-11 and (B.i) IL-6 protein, or (A.ii) *IL11* or (B.ii) *IL6* mRNA was measured by ELISA or qPCR, respectively. In a separate experiment, EC were exposed to various cytokines for 4 h, followed by supernatant collection and measurement of (A.iii) IL-11 and (B.iii) IL-6. ‘Max hCMV’ is the maximal induction induced by hCMV, as a comparison. Data are means ± SEM of 3–5 experiments (all using EC from independent donors) ^*^p < 0.05 ^**^p < 0.01 ^***^p < 0.001 vs. mock-inoculated, or untreated.

EC were mock-inoculated, or treated with a clinical strain of hCMV (VR1814) at a multiplicity of infection (MOI) of 5, which gave the highest percentage infection at 24 h in the absence of cell death, determined by trypan blue exclusion. Supernatants were collected from each 24 h period over 7 days and EC lysates were collected at day 1, 3, 5 and 7 post inoculation (dpi). IL-11 and IL-6 secreted protein or transcript expression was measured by ELISA or qPCR, respectively. Secreted IL-11 was not detected in supernatants from mock-inoculated EC at any time point tested Fig. 1(A.i). hCMV induced IL-11 secretion by 2dpi (mean [pg/ml] ±SEM: 243 ± 60) and levels continued to increase in magnitude up to 7dpi (mean [pg/ml] ±SEM: 2290 ± 469) (Fig. 1A.i). *IL11* mRNA transcription was induced by hCMV by 1dpi (mean [fold increase] vs. mock ± SEM: 14.1 ± 2.4), leading to a peak at 5dpi (mean [fold increase] vs. mock ± SEM: 107.3 ± 51.3) (Fig. 1A.ii). Previous studies have shown that respiratory syncytial virus and parainfluenza virus type 3/rhinovirus infection induce IL-11 production from antigen presenting cells [1] and stromal cells [4], respectively. However, this is the first report of any virus, or indeed biological stimulus, that induces EC IL-11 production. We could not detect EC IL-11 in supernatants following EC stimulation with IL-1, LPS, TNFα, IFNγ or IFNβ (Fig. 1A.iii) or cell stress induced by H_2_O_2_, staurosporine or serum starvation (data not shown), suggesting that hCMV-induced IL-11 induction was not a consequence of a general inflammatory or cellular stress response. Previous reports of IL-1 stimulated smooth muscle cells producing IL-11 [5], indicate that cell-type specific regulation exists.

IL-6 protein was secreted by mock-inoculated EC (Fig. 1B.i), as previously reported [11]. hCMV infection enhanced IL-6 secretion by 1dpi (mean [pg/ml] ±SEM: hCMV vs. mock, 1733 ± 91.7 vs. 1331 ± 112), which peaked at 4dpi (mean [pg/ml] ±SEM: hCMV vs. mock, 2540 ± 481.2 vs. 577 ± 94), before returning to baseline by 7dpi (Fig. 1B.i). *IL6* mRNA was increased by hCMV by 1dpi (mean [fold increase] ±SEM vs. mock 6.5 ± 2.6) and this elevation returned to baseline by 5dpi (Fig. 1B.ii). For both IL-11 and IL-6, there was a temporal delay between changes in mRNA level and expression of the corresponding protein, as is often observed during dynamic adaptation processes [13]. Unlike IL-11, IL-6 protein was secreted in response to a range of cytokines (Fig. 1B.iii), highlighting further independent regulatory programmes.

**Fig. 2:**
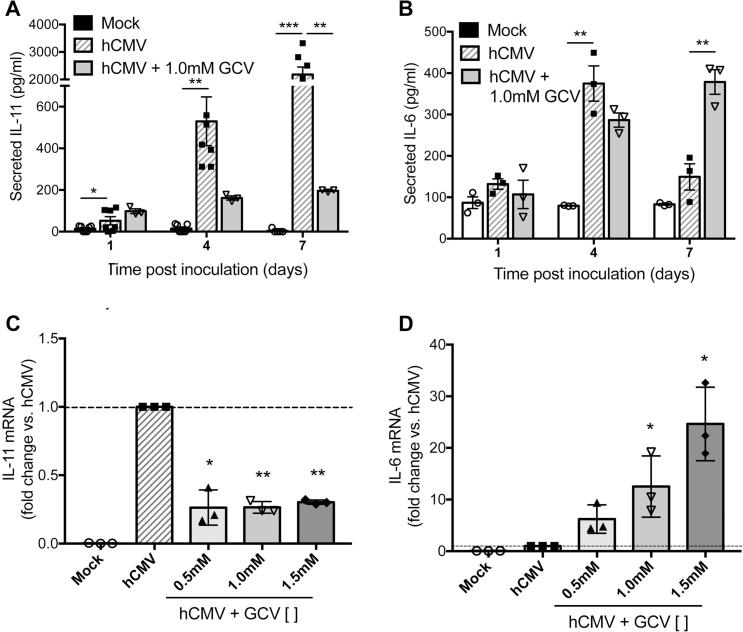
Human cytomegalovirus induced EC IL-11 and IL-6 expression is differentially modulated by viral gene expression. EC were mock- or hCMV-inoculated and treated with or without ganciclovir (GCV) at the concentrations stated. Supernatants were collected over a 24 h period at 1, 4 or 7 days post inoculation (dpi), and secreted (A) IL-11 or (B) IL-6 protein was measured by ELISA. EC were harvested at 7dpi and analysed for mRNA expression of (C) IL-11 or (D) IL-6. GCV: Ganciclovir. Data are means ± SEM of 3–6 experiments (all using EC from independent donors) ^*^p < 0.05 ^**^p < 0.01 ^***^p < 0.001.

The variation in expression dynamics of hCMV-induced IL-11 and IL-6 could indicate differential regulation of these cytokines during the hCMV life cycle, which has distinct stages. hCMV ‘immediate early’ genes are expressed within hours of infection, followed by ‘delayed early’ genes, which initiate the process of viral genome replication [10]. ‘Late’ gene expression follows, which initiates capsid assembly and egress to the cytoplasm. hCMV-inoculated EC express proteins encoded by early, but not late, hCMV genes at 1dpi, with the majority (>60%) of cells being positive for late proteins by 7dpi [11]. To explore the effect of viral replication on EC IL-11 and IL-6, we cultured mock- or hCMV-inoculated EC with or without 1.0 mM ganciclovir (GCV), which inhibits the synthesis of viral DNA. Untreated and ganciclovir treated EC remained equally viable, comparable in number and baseline production of secreted IL-11 and IL-6 were not modulated by the presence of the drug at any time point (data not shown). Real time PCR (qPCR) was used to confirmed that ganciclovir inhibited the expression of hCMV late genes that encode for structural proteins, glycoprotein B and pp65, by >98% at 7dpi (mean reduction [%] ±SEM: 98.7 ± 0.4 and 99.7 ± 0.005, respectively). Supernatants were collected from 24 h periods starting at 1, 4 and 7 dpi. hCMV induced IL-11 secretion by 4dpi, with further increase by 7dpi (Fig. 2A). This induction was markedly inhibited in the presence of ganciclovir (hCMV vs. hCMV + GCV [pg/ml] ±SEM: 4dpi 529 ± 117 vs. 161 ± 10, 7dpi 2177 ± 276 vs. 201 ± 2.9) (Fig. 2A), indicating that it was dependent on viral DNA synthesis. Conversely, hCMV-induced IL-6 peaked at 4dpi and was unaffected by ganciclovir (hCMV vs. hCMV + GCV [pg/ml] ±SEM: 4dpi 374 ± 42 vs. 286 ± 17) (Fig. 2B). hCMV-induced IL-6 returned to near baseline levels by 7dpi, but the presence of ganciclovir prevented this reduction, and levels remained comparable to the peak observed at 4dpi (hCMV vs. hCMV + GCV [pg/ml] ±SEM: 7dpi 149 ± 32 vs. 378 ± 30). To determine if these effects were due to transcriptional or post-transcriptional modifications we measured the effect of ganciclovir on hCMV-induced *IL11* and *IL6* mRNA at 7dpi. hCMV-induced *IL11* mRNA was significantly inhibited by 1.0 mM ganciclovir (mean inhibition [%] ±SEM: 73.7 ± 2.3) (Fig. 2C), with comparable inhibition observed with 0.5 and 1.5 mM ganciclovir. Conversely, *IL6* mRNA was elevated by 1.0 mM ganciclovir (mean increase 1.0 mM GCV [%] ±SEM: 1250 ± 340) (Fig. 2D), and this response was dose dependent (ANOVA (dose) p > 0.002) (Fig. 2D). Thus, the effects of ganciclovir on hCMV regulation of IL-11 and IL-6 secretion were mediated at the transcriptional level.

These data provide the first description of opposing effects of hCMV gene expression on EC IL-11 and IL-6 production, i.e. the *induction* of EC IL-11 secretion and concurrent *suppression of* EC IL-6. The consequence of hCMV-induced IL-11 production is unknown, but recombinant IL-11 conferred protection from apoptosis to EC [17], [14] and thus, one could speculate it could convey a hCMV survival advantage during replication. Furthermore, IL-11 can stimulate the growth of CD34 positive myeloid lineage cells [12], which constitute a major site for hCMV latency. Thus, virus induced IL-11 production could enhance the pool of latently infected cells. Previous studies showed that hCMV infection of fibroblasts initially induced IL-6 transcription, but that this induction was suppressed when productive infection occurred [7]. This is in alignment with our observations that GCV-mediated suppression of hCMV replication enhanced IL-6 production. The suppression of EC IL-6 could provide a viral survival advantage suppressing inflammation, or other downstream effects of IL-6. It would be interesting to study the relationship between hCMV viral life cycle and IL-6 regulation in other susceptible cell types, to determine if this is a general hCMV mechanism.

In conclusion, we report that hCMV induces the transcription and subsequent secretion of EC IL-11, and that this is linked to the onset of viral replication.

## 3 Methods

### 3.1 hCMV production

The hCMV clinical strain, VR1814 (gift from Dr. Giuseppe Gerna, University of Pavia, Italy) was propagated in human umbilical vein EC (HUVEC), as described [8]. Virus titre (infectious units/ml) was determined by plaque assay.

### 3.2 Isolation, culture and inoculation of human umbilical vein endothelial cells

HUVEC were isolated from umbilical cords and validated as >96% pure, as previously described [3], and cultured in Medium 199 (M199, Gibco) with 20% foetal bovine serum (FBS), 100U/ml penicillin, 0.1 mg/ml streptomycin, 1 ng/ml human epidermal growth factor (Sigma), and 1.25 μg/ml Amphotericin B (Invitrogen). HUVEC were cultured to a confluent monolayer prior to hCMV infection/treatment with ganciclovir. Each experiment used a separate donor. EC were inoculated with hCMV at a multiplicity of infection of 5 (viral particles/cell). Inoculated EC were incubated for 90 min at 37 °C before the medium was replaced. Infection was confirmed at 24 h Supernatants/EC were harvested as appropriate.

### 3.3 Evaluation of IL-11 and IL-6 secreted protein

IL-11 or IL-6 protein was measured in EC supernatants using commercial ELISA kits (Human IL-11 or IL-6 DuoSet ELISA, R&D Systems).

### 3.4 Evaluation of human *IL11* and *IL6* and hCMV pp65 and glycoprotein B mRNA

Total RNA was isolated using the RNeasy Mini kit with QIAshredder (Qiagen). RNA was converted to cDNA using the SuperScript III First-Strand Synthesis System with Oligo(dT) 20 or random hexamers (Invitrogen, Life Technologies) according to the manufacturer's instructions. Human *IL11* or *IL6* and hCMV pp65 and glycoprotein B mRNA was measured using the TaqMan Fast Universal PCR Master Mix with the relevant Applied Biosystems TaqMan MGB primers/probe mix. 18S was used as an endogenous control (Applied Biosystems, Life Technologies). A 7900HT Fast Real-Time PCR system (Applied Biosystems) was used and data were analysed using SDS 2.4 software. Relative expression was calculated by the comparative CT method.

### 3.5 Statistics

Data was analysed using paired or unpaired *t-test*, as appropriate, or ANOVA (Prism).

## Funding

This work was supported by The Swedish Research Council (Vetenskapsrådet) [2013–42608–102305–28] awarded to LB. TR acknowledges support from the German Research Society (SFB877, TP A11 and SFB841, TP B8), and a European Research Council grant (ERC-StG-2012-311575_F-12).

## Competing interests

The authors have no competing interests to declare.
